# The potential of mRNA expression evaluation in predicting HER2 positivity in gastroesophageal cancer

**DOI:** 10.1590/1414-431X2022e12428

**Published:** 2022-11-11

**Authors:** I.M. de Oliveira, P. Nicolau-Neto, P.V. Fernandes, T.S. Lavigne, P.F. Neves, J.C. Tobar, S.C. Soares-Lima, T.A. Simão, L.F. Ribeiro Pinto

**Affiliations:** 1 Divisão de Patologia, Instituto Nacional de Câncer, Rio de Janeiro, RJ, Brasil; 2 Programa de Carcinogênese Molecular, Instituto Nacional de Câncer, Rio de Janeiro, RJ, Brasil; 3 Departamento de Bioquímica, Universidade do Estado do Rio de Janeiro, Rio de Janeiro, RJ, Brasil

**Keywords:** Gastroesophageal carcinoma, HER2, mRNA expression

## Abstract

Gastroesophageal cancer (GEC) is an aggressive disease characterized by a high frequency of metastasis and poor overall survival rates. GEC presents HER2 overexpression in 5 to 25% of tumors eligible for HER2-targeted therapy. HER2 evaluation requires protein levels and copy number alteration analyses by immunohistochemistry (IHC) and *in situ* hybridization (FISH or SISH), respectively. These are semiquantitative methodologies that need an expert and well-trained pathologist. Therefore, the use of new surrogate methods for HER2 evaluation in cancer, such as gene expression analysis, might improve GEC HER2 classification. We evaluated HER2 positivity in GEC through conventional IHC and SISH analyses and investigated the potential application of HER2 mRNA expression by quantitative PCR to categorize GEC samples as HER2-positive or HER2-negative. Among 270 GEC samples, 10.9% were HER2-positive by IHC and SISH analyses. HER2 mRNA was overexpressed in HER2-positive GEC samples and presented high accuracy in distinguishing those tumors from HER2-negative GEC. Nevertheless, HER2 mRNA analysis was not capable of classifying HER2-equivocal GEC samples into HER2-positive or -negative according to SISH data. Quantitative PCR analysis showed HER2 overexpression in HER2-positive GEC samples. Nevertheless, HER2 mRNA analysis failed to classify HER2-equivocal GEC according to SISH data.

## Introduction

Gastroesophageal cancer (GEC) is a significant health problem worldwide, with more than 1.5 million new cases and 1.2 million deaths annually (1). GEC is an aggressive disease, with a high frequency of lymph node and distant metastasis upon its diagnosis (2,3) and overall survival rates of 5-20%, making GEC the third most lethal tumor worldwide (1,4,5).

Advances in the molecular classification of GEC highlighted the potential implementation of targeted therapies, including a subgroup of GEC with the Human Epidermal growth factor Receptor 2 (*HER2* - *ERBB2* gene) overexpression (6-8). HER2 is an essential driver in several tumors, associated with cell proliferation, migration, and differentiation (9). In addition, HER2 has been validated as a prognostic and predictive factor in breast cancer (10), and there is growing evidence for a role of HER2 in GEC tumorigenesis, with studies frequently reporting *HER2* amplification or overexpression in this tumor (6-9).

HER2 targeted therapy is the only approved membrane-bound receptor tyrosine kinase (RTK) treatment for GEC patients. The Trastuzumab for Gastric Cancer study (ToGA) revealed 22% of HER2-positive GEC, but HER2 positivity frequency differed according to the localization and histologic classification, with esophagogastric junction tumors (EGJ) and gastric tumors (GC) presenting 32.2 and 21.4% of positivity, respectively (9). Additionally, the ToGA study revealed a significant prognosis improvement in patients with HER2-positive advanced-stage gastric cancer treated with chemotherapy and trastuzumab, a HER2-targeting monoclonal antibody (6,8).

HER2-positive tumors have been initially described in breast cancer and more recently in GEC using immunohistochemistry (IHC), with further fluorescence *in situ* hybridization (FISH) or silver *in situ* hybridization (SISH) for those classified as uncertain to verify *HER2* gene amplification (11). However, some authors have demonstrated other mechanisms regulating *HER2* expression with an impact on patient prognosis, besides gene amplification (12-15). Thus, evaluation of *HER2* mRNA expression in GEC could add information to support patient stratification for targeted therapy.

Therefore, in this work, we evaluated HER2-positive GEC through conventional IHC and SISH and compared it to *HER2* mRNA expression by quantitative polymerase chain reaction (PCR), analyzing the impact of HER2-positive cases on prognosis and other clinicopathological characteristics of Brazilian GEC patients.

## Material and Methods

### Samples

This was a retrospective study that included 270 GEC cases diagnosed by histopathological examination according to the Union for International Cancer Control and the American Joint Committee on Cancer (UICC/AJCC, 7th edition). Patients were submitted to curative surgical resection, with or without adjuvant therapy, at the Instituto Nacional de Câncer (INCA, Brazil) from January 1999 to December 2006. The results of HER2 expression were generated after the patients were treated and, therefore, did not guide treatment choice. None of the patients received HER2-targeted therapy. Clinicopathological data of patients were obtained through their medical records. The Ethics Committee of the institution approved this project under protocol number 134/11, and patients signed a consent form to allow the use of samples and data records in the study.

### HER2 protein evaluation by immunohistochemistry

HER2 protein expression was assessed by immunohistochemistry. Tissue slides of 3 to 4 µm thickness were prepared from formalin-fixed paraffin-embedded (FFPE) GEC blocks, and the immunoreaction was performed with an anti-HER2/neu monoclonal antibody (clone 4B5), followed by ultraView DAB Revelation Kit (Roche, Switzerland), according to the manufacturer's recommendations. The criteria for the HER2 interpretation score system established by Hofmann et al. (11) was used: negative result, score 0, no reactivity or membrane reactivity in less than 10% of neoplastic cells; negative result, score 1+, weak or incomplete membrane reactivity in 10% or more neoplastic cells (reactive cells in only part of the membrane); doubtful or suspicious result, score 2+, complete to basolateral or lateral weak to moderate membrane reactivity in 10% or more of neoplastic cells; positive result, 3+ score, was complete strong basolateral or lateral membrane reactivity in 10% or more of neoplastic cells (11,16,17).

### Dual-color SISH analysis

For dual-color SISH, 5-mm-thick sections from FFPE tissue blocks were prepared. Dual-color SISH slides were also processed using an automated system that followed the manufacturer's protocols for HER2 DNA and chromosome 17 probe using the BENCHMARK XT (Ventana Medical Systems-Roche Group, USA). Both probes were sequentially hybridized in one slide. A single copy of the *HER2* gene is visualized as a black dot. A red dot for chromosome 17 appears following the reaction with fast red and naphthol phosphate. We interpreted the results using the American Society of Clinical Oncology/College of American Pathologists (ASCO/CAP) guidelines for all three methods (18).

### *HER2* mRNA expression evaluation by qPCR

*HER2* gene expression was analyzed in GEC using 13 randomly selected samples classified as HER2-negative (IHC 0/1+), all of the 25 samples classified as equivocal (IHC 2+), and all of the 21 HER2-positive samples (IHC 3+). RNA was extracted using the PureLink FFPE RNA isolation kit (Thermo, USA), following the manufacturer's protocol. cDNA was synthesized with 500 ng of total RNA using SuperScript III (Thermo).

The gene expression analysis was performed on the Rotor-Gene (Qiagen, Germany) real-time PCR platform using the TaqMan Universal PCR Fast Master Mix (Applied Biosystems, USA) with *GAPDH* (Hs02786624_g1) and *HER2* (HER2, Hs01001580_m1) TaqMan Gene Expression Assays (Applied Biosystems). A standard normalized fluorescence threshold of 0.1 was set to identify cycle threshold (CT) values. The difference between the means of three experiments of the gene of interest (*HER2*) and the reference gene (*GAPDH*) was calculated with Microsoft Excel software (USA), and the relative quantification value was expressed as 2^-ΔCt (19). Samples with no *GAPDH* amplification in PCR were removed from this analysis. Therefore, ten samples were excluded from qPCR analysis (two HER2-negative, three HER2-equivocal, and five HER 2-positive).

### Statistical analyses

Statistical analyses were performed with GraphPad Prism 5.0 (USA). We used Fisher's exact test to assess the relationship between *HER2* amplification and expression and clinicopathological features. The Kolmogorov-Smirnov test was applied to verify the Gaussian distribution for gene expression analysis. Kruskal-Wallis test with Dunn's post-test was applied to verify *HER2* mRNA expression differences in GEC according to HER2 protein status. The final values were considered of statistical significance when P<0.05. The ROC curve was performed for the identification of *HER2* mRNA expression accuracy to predict gene amplification in GEC. The Kaplan-Meier method and log-rank test were used for survival analyses. Variables with P<0.2 were selected for multivariate analysis. Finally, Cox regression was applied with the stepwise forward method. These analyses were performed in the R environment using package survival.

## Results

### Profile of patients with GEC

A total of 270 patients with GEC were included (mostly men, 57.4%), with a median age at diagnosis of 61 years (range 29-88 years). Most cases were diagnosed as Lauren's intestinal histological subtype (n=115; 42.6%). Most tumors were identified in the stomach (GC, n=226; 83.7%), compared with tumors arising at the EGJ. Most patients (n=156, 57.8%) had advanced disease, presented lymph node metastasis (cN+; 50.4%), and perineural (n=153; 56.7%) but no vascular invasion (n=242, 89.6%) (Table 1).

**Table 1:** Clinicopathological features of gastroesophageal cancer samples.

Variables	Frequency	
	n	%
Age (years)		
<50	60	22.2
≥50	210	77.8
Gender		
Male	155	57.4
Female	115	42.6
Skin color		
White	182	67.4
Non-white	88	32.6
Alcohol Drinking		
No	138	51.1
Yes	119	44.1
NA	13	4.8
Tobacco Smoking		
No	109	40.4
Yes	153	56.7
NA	8	3.0
*H. pylori* infection history		
Yes	139	51.5
No	127	47.0
NA	4	1.5
Lauren's classification		
Intestinal type	115	42.6
Diffuse type	106	39.3
Mixed type	49	18.1
Lymph node metastasis		
No	134	49.6
Yes	136	50.4
Vascular invasion		
No	242	89.6
Yes	28	10.4
Perineural invasion		
No	117	43.3
Yes	153	56.7
Tumor Stage		
Early (I/II)	114	42.2
Late (III/IV)	156	57.8
Tumor Relapse		
No	254	94.1
Yes	16	5.9
Metastasis		
No	194	71.9
Yes	76	28.1

### HER2 status in GEC

**Figure 1:**
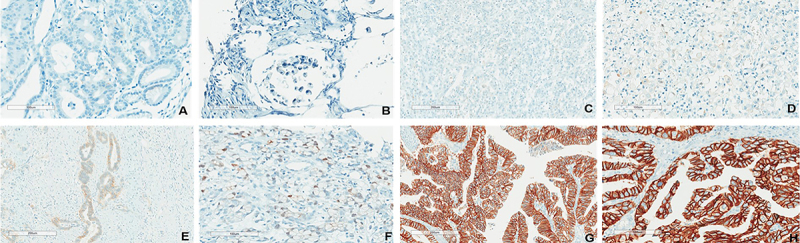
Representative images of HER2 protein expression in gastroesophageal cancer by immunohistochemistry. **A** and **B**, Negative result, score 0, showing no reactivity in neoplastic cells (**A** 100×, **B** 200×, scale bars 200 and 100 μm); **C** and **D**, Negative result, score 1+, showing weak or incomplete membrane reactivity in 10% or more neoplastic cells (**C** 100×, **D** 200×, scale bars 200 and 100 μm); **E** and **F**, Equivocal result, score 2+, showing complete to basolateral or lateral weak to moderate membrane reactivity in 10% or more of neoplastic cells (**E** 100×, **F** 200×, scale bars 200 and 100 μm); **G** and **H**, Positive result, 3+ score, showing complete strong basolateral or lateral membrane reactivity in 10% or more of neoplastic cells negative samples (**G** 100×, **H** 200×, scale bars 200 and 100 μm).

Immunohistochemical (IHC) analyses were performed in 270 surgical samples and revealed 26 cases overexpressing HER2 (3+; 9.63%), 25 equivocal samples (2+; 9.26%), and 219 HER2-negative samples (81.11%), 15 of which (5.55%) had score 1+ (Figure 1).

To elucidate if equivocal HER2-2+ GEC had *HER2* gene amplification, we carried out SISH analysis in 11 out of 25 samples (fourteen samples did not show the required quality to perform this analysis). Among these, seven showed *HER2/*chr17 centromere ratio ≥2 (range 2.00-2.75) and four were classified as not amplified (*HER2*/chr17 centromere ratio <2, range 0.8-1.5). Therefore, among 256 samples with enough quality to analyze *HER2* status (94.8% of total), 33 (12.9%) GEC were classified as HER2-positive.

### *HER2* mRNA expression evaluation in GEC samples

**Figure 2:**
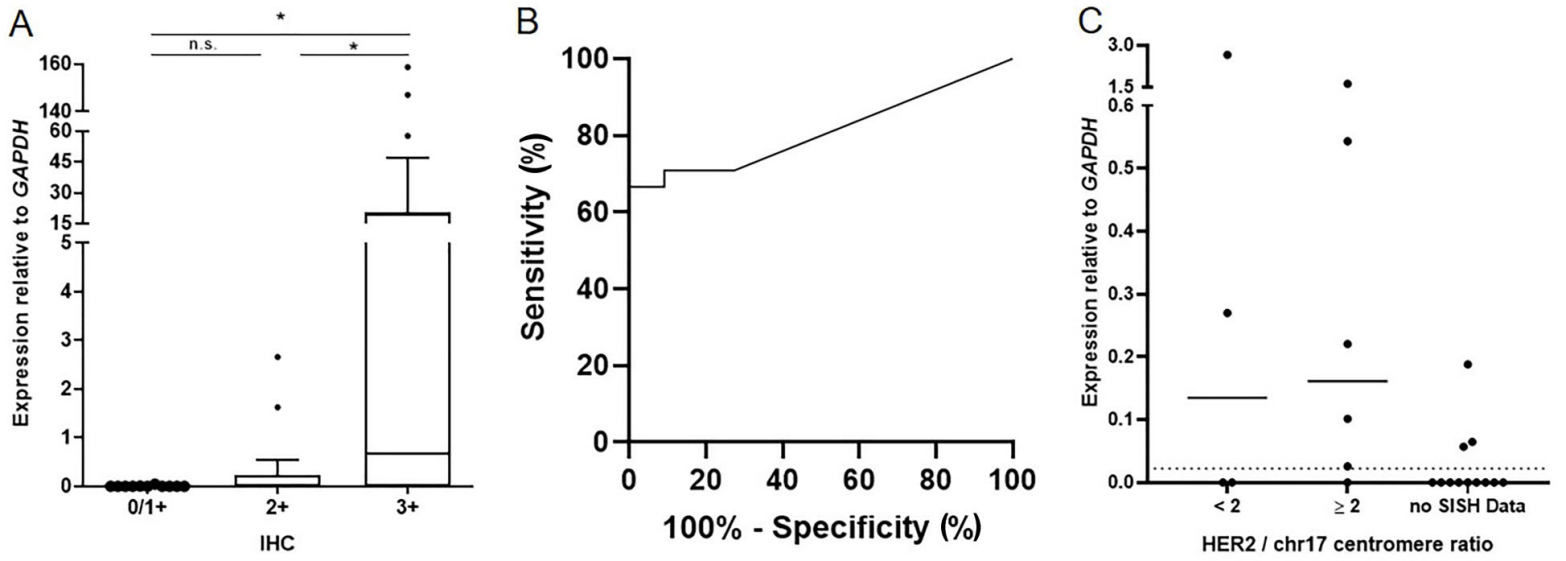
*HER2* gene expression profile in gastroesophageal cancer (GEC) according to HER2 protein levels. **A**, *HER2* mRNA was overexpressed in GEC with HER2 immunohistochemistry (IHC) 3+ score compared to HER2-negative samples (IHC 1+/0) and HER2-equivocal samples. Data are reported as means±SD, *P<0.05 (Kruskal-Wallis test with Dunn's post-test). **B**, Receiver operating characteristics (ROC) curve analysis showing 70.8% of sensitivity and 90.9% of specificity from *HER2* expression to discriminate HER2 IHC 3+ GEC samples to HER2-negative samples (P=0.003). **C**, *HER2* mRNA was not differentially expressed between HER2 equivocal samples with or without HER2 amplification by silver *in situ* hybridization (SISH) (P=0.91). Among 14 equivocal samples without SISH data, three presented *HER2* mRNA expression similar to HER2-positive GEC. The dotted line represents the *HER2* expression cut-off identified in the ROC curve analysis.

The *HER2* (*ERBB2*) mRNA expression was assessed in GEC samples according to their HER2 IHC classification status (Figure 2A). Among 13 HER2-negative samples (IHC 0/1+) analyzed by PCR, two were excluded, eight showed no *HER2* expression, and three samples showed *HER2* expression. In all 25 HER2-equivocal samples (IHC 2+), three were excluded from the analysis, 12 showed no *HER2* mRNA expression, and 10 showed *HER2* expression. Among HER2-positive samples (IHC 3+), 16 showed *HER2* expression in the PCR analysis, five showed no *HER2* expression, and five were excluded from the analysis. Samples with no *GAPDH* amplification by qPCR were removed from the analysis.

*HER2* mRNA was overexpressed in HER2-positive samples compared with both HER2-negative samples (mean fold-change=4,447) and HER2-equivocal samples (mean fold-change=90.7) (P=0.003). However, no expression difference was observed between negative and equivocal samples.

*HER2* mRNA expression level distinguished HER2-positive GEC (IHC 3+) from HER2-negative (IHC 0/1+) with 81.6% accuracy using as cut-off an expression value relative to *GAPDH* of 0.02210 (P=0.003), conferring 70.8% of sensitivity and 90.9% of specificity (Figure 2B).

In order to evaluate if *HER2* expression analysis by quantitative PCR could be an alternative method to HER2 SISH analysis in GEC, we compared the *HER2* gene expression profile in equivocal samples with SISH data. Although 14 of 25 equivocal samples did not have quality for SISH analysis, 11 samples were included in the gene expression evaluation. However, no *HER2* gene expression difference was observed in equivocal samples according to the HER2-SISH status (P=0.91) (Figure 2C).

### HER2 status and clinicopathological features

No association was observed between HER2-positivity and clinicopathological data (Table 2). In comparing the features of GEC from GC or EGJ, differences were observed in sex (P=0.003), alcohol drinking (P=0.037), Lauren's classification (P=0.02), vascular invasion (P=0.007), tumor stage (P<0.001), and presence of metastasis (P=0.0017) (Supplementary Table S1).

**Table 2:** Association between HER2 status and clinicopathological features of gastroesophageal cancer.

	Frequency		HER2 positivity		
Variables	n^#^	%	Yes	No	P
Age (years)					
<50	58	22.7	7	51	0.99
≥50	198	77.3	26	172	
Gender					
Male	144	56.2	19	125	0.99
Female	112	43.8	14	98	
Skin color					
White	171	66.8	26	145	0.16
Non-white	85	33.2	7	78	
Alcohol Drinking					
No	131	51.1	17	114	0.99
Yes	112	43.8	15	97	
NA	13	5.1	1	12	
Tobacco smoking					
No	104	40.6	15	89	0.57
Yes	144	56.3	17	127	
NA	8	3.1	1	7	
*H. pylori* infection history					
Yes	121	47.2	18	103	0.25
No	131	51.2	13	118	
NA	4	1.6	2	2	
Lauren's classification					
Intestinal type	109	42.6	17	92	0.12*
Diffuse type	103	40.2	8	95	0.34**
Mixed type	44	17.2	8	36	0.056***
Lymph node metastasis					
No	126	49.2	14	112	0.45
Yes	130	50.8	19	111	
Vascular invasion					
No	231	90.2	29	202	0.54
Yes	25	9.8	4	21	
Perineural invasion					
No	113	44.1	19	94	0.13
Yes	143	55.9	14	129	
Tumor stage					
Early (I/II)	108	42.2	10	98	0.18
Late (III/IV)	148	57.8	23	125	
Tumor relapse					
No	244	93.3	32	212	0.99
Yes	12	4.7	1	11	
Metastasis					
No	185	72.3	23	162	0.68
Yes	71	27.7	10	61	

256 samples with immunohistochemistry or silver *in situ* hybridization analyses. *Comparison of the three Lauren's classification types independently; **intestinal type compared to diffuse plus mixed type; ***intestinal plus mixed types compared to diffuse type (Fisher's exact test).

The prognostic significance of HER2 expression in GEC was also evaluated. Patients with HER2-positive tumors presented a median survival of 30.7 months, while patients with HER2-negative GEC exhibited a median survival of 52.7 months, but this difference was not significant (HR=1.01; P=0.96; 95%CI: 0.65-1.57). Univariate survival analyses of clinicopathological characteristics indicated lymph node invasion (HR=2.5; P=9.7e-9; 95%CI: 1.83-3.43), perineural invasion (HR=2.01; P=1.7e-5; 95%CI: 1.46-2.77), late-stage tumor (HR=4.23; P=5.4e-15; 95%CI: 2.94-6.07), and tumor located in EGJ (HR=2.22; P=3.9e-5; 95%CI: 1.51-3.22) as features associated to worse prognosis. Multivariate analysis revealed lymph node invasion (HR=1.49; P=0.02; 95%CI: 1.05-2.09) and late-stage (HR=3.05; P=1.5e-7; 95%CI: 2.01-4.63) as independent prognostic factors (Table 3) (Supplementary Figure S1).

**Table 3:** Survival analysis of prognostic factors for gastroesophageal cancer.

	Univariate analysis			Multivariate analysis		
Variables	HR	95%CI	P value	HR	95%CI	P value
Age (years)						
≥50 *vs* <50	1.23	0.83-1.81	0.28			
Gender						
Female *vs* Male	0.87	0.64-1.19	0.39			
Skin color						
Non-white *vs* white	0.92	0.66-1.28	0.64			
Alcohol drinking						
Current/former *vs* never	0.99	0.72-1.36	0.98			
Tobacco smoking						
Current/former *vs* never	1.15	0.83-1.58	0.38			
*H. pylori* infection						
Yes *vs* no	1.12	0.82-1.53	0.45			
Lauren's classification						
Diffuse and mixed *vs* intestinal	0.99	0.71-1.34	0.96			
Lymph node invasion						
Yes *vs* no	2.5	1.83-3.43	**9.75E-09**	1.49	1.05-2.09	**0.02**
Vascular invasion						
Yes *vs* no	1.37	0.86-2.19	0.18			
Perineural invasion						
Yes *vs* no	2.01	1.46-2.77	**1.79E-05**	1.27	0.90-1.78	0.15
Tumor stage						
Late (III/IV) *vs* Early (I/II)	4.23	2.94-6.07	**5.43E-15**	3.05	2.01-4.63	**1.55E-07**
Tumor site						
Gastric *vs* EGJ	0.45	0.31-0.66	**3.95E-05**	0.73	0.49-1.08	0.11
HER2 amplification						
Yes *vs* no	1.01	0.65-1.57	0.96			

HR: Hazard ratio; CI: confidence interval. Bold type indicates statistically significant.

Furthermore, association analyses between *HER2* mRNA expression and GEC clinicopathological features were performed, and only tumor size was associated with *HER2* expression, with larger tumors (≥5 cm) showing 10-fold higher *HER2* expression than smaller tumors (<5 cm) (95%CI: 1.54-62.67; P=0.02).

## Discussion

We performed an extensive study on the HER2 profile in GEC samples of patients treated at a large Brazilian public oncology institution, and evaluated gene expression, protein level, and gene amplification by SISH. Our findings showed 12.9% of HER2-positive in a GEC set composed of both early and advanced tumors, in concordance with the HER2-positive GEC frequency range shown in different studies worldwide (5 to 25%) (8,20,21). In addition, we demonstrated differences in *HER2* mRNA expression according to IHC status in GEC. Nevertheless, the *HER2* mRNA level did not show high accuracy to classify HER2- equivocal GEC according to SISH classification.

This study showed a higher frequency of HER2-positive GC cases than other studies evaluating HER2 status (HER2-positive samples ranged from 4.7 to 10.5%) in Brazilian patients (22-24). This fact may be explained by the higher percentage of EGJ tumors included in our study, since the frequency of HER2-positive tumors is higher in the EGJ than in the stomach (8).

We showed that *HER2* mRNA is overexpressed in GEC HER2-positive samples compared with negative or equivocal GEC samples. Nevertheless, *HER2* mRNA expression evaluation did not show high agreement with SISH classification to classify HER2-equivocal GC cases. These results are similar to those of breast cancer studies. *HER2* expression was lower in HER2-negative breast cancer samples and higher in HER2-positive samples (25). However, the agreement between IHC and gene expression analysis by quantitative PCR data was limited in HER2-equivocal and -positive breast cancer samples, with an agreement of only 67%. *HER2* mRNA expression evaluation was previously reported in GC. Ma et al. (26) showed that GC presented higher *HER2* expression than peritumoral tissue, and advanced tumors showed higher expression than early-stage tumors. The present study seems to be, so far, the only one applying mRNA analysis to classify HER2-equivocal GEC samples according to SISH data.

Other molecular mechanisms have already been described as regulators of HER2 expression, such as transcription factors (TFs) activity (27,28). TFs can act as a multifunctional coactivator/corepressor complex of the *HER2* expression in cancer (29). The transcription factors YY1 and AP-2 were previously associated with *HER2* mRNA expression in breast cancer samples without *HER2* amplification (11). YY1 was already described as overexpressed in GC and associated with worse prognosis (12,13). AP-2 overexpression was associated with a better prognosis in GC, inhibiting the Notch signaling pathway (14,15). Notch and HER2 pathways work together in the resistance to trastuzumab therapy. Breast cancer HER2+ cells with acquired resistance to an anti-HER2 therapy showed overexpression of *Notch-1* and its canonical target genes (30,31). The overexpression of all Notch receptors was associated with a worse prognosis in GEC, both in HER2-negative and HER2-positive tumors (32).

Recently, *HER2* expression was associated with epigenetic events in breast cancer cells. Histone modifications, protein binding, and DNA hypermethylation into *HER2* gene body enhancers were related to HER2 expression (27). There are no data regarding histone modification and DNA methylation regulation of *HER2* expression in GEC so far. Nevertheless, the microRNA miR-204-5p activity was associated with *HER2* downregulation, resulting in inhibition of cell proliferation, migration, and invasion and promoting apoptosis in GC cells (33).

In our study, *GAPDH* expression was used as an mRNA integrity filter in qPCR analysis, including only samples with mRNA integrity in the quantitative PCR analysis. Although *HER2* mRNA was overexpressed in HER2 IHC 3+ GEC, some HER2-positive samples did not show *HER2* mRNA expression. Nevertheless, there are other reasons that could explain a lack of correlation between *HER2* mRNA overexpression and protein expression in some HER2 IHC 3+ samples. In general, stimulation with ligands induces activation of RTKs, autophosphorylation, and recruitment of c-Cbl, a member of the Cbl family of ubiquitin-protein ligases. c-Cbl is then phosphorylated and ubiquitylates the RTK directing it to endocytosis and degradation (34). Previous studies reported that HER2 could be resistant to endocytosis and degradation due to active retention in the cell membrane or lack of internalization signals in cancer cells (35). Among RTKs, HER2 presents a lower affinity to c-Cbl (36), and this is related to the maintenance of HER2 protein overexpression. In addition, HER2 localizes in caveolae domains (37). Caveolae are caveolin-1 enriched subdomains of the cell membrane (38), and modulation of the caveolae domains could promote the stability of HER2 at the cell membrane (39). Some studies showed that only 15 to 20% of GC samples have a caveolin-1 expression (40). These features could explain the molecular mechanisms behind the presence of protein overexpression in GEC samples with low *HER2* gene expression. Nevertheless, other studies need to be performed to test these hypotheses.

A limitation of this study was the use of FFPE samples, which might impact *HER2* gene expression analysis due to the deleterious effects of formalin fixation on RNA integrity. Therefore, other studies using *HER2* mRNA in a set of fresh-frozen or fresh FFPE GEC samples should be performed to validate our data.

In conclusion, quantitative PCR analysis showed HER2 overexpression in HER2-positive GEC samples. Nevertheless, *HER2* mRNA analysis failed to classify HER2-equivocal GEC according to SISH data.

## Supplementary Material

Click here view [pdf].
